# Vitamin D Deficiency and Exogenous Vitamin D Excess Similarly Increase Diffuse Atherosclerotic Calcification in Apolipoprotein E Knockout Mice

**DOI:** 10.1371/journal.pone.0088767

**Published:** 2014-02-19

**Authors:** Timothy Ellam, Abdul Hameed, Risat ul Haque, Munitta Muthana, Martin Wilkie, Sheila E. Francis, Timothy J. A. Chico

**Affiliations:** 1 Department of Cardiovascular Science, University of Sheffield, Sheffield, United Kingdom; 2 Sheffield Kidney Institute, Northern General Hospital, Sheffield, United Kingdom; 3 Department of Infection and Immunity, University of Sheffield, Sheffield, United Kingdom; UAE University, Faculty of Medicine & Health Sciences, United Arab Emirates

## Abstract

**Background:**

Observational data associate lower levels of serum vitamin D with coronary artery calcification, cardiovascular events and mortality. However, there is little interventional evidence demonstrating that moderate vitamin D deficiency plays a *causative* role in cardiovascular disease. This study examined the cardiovascular effects of dietary vitamin D deficiency and of vitamin D receptor agonist (paricalcitol) administration in apolipoprotein E knockout mice.

**Methods:**

Mice were fed atherogenic diets with normal vitamin D content (1.5IU/kg) or without vitamin D. Paricalcitol, or matched vehicle, was administered 3× weekly by intraperitoneal injection. Following 20 weeks of these interventions cardiovascular phenotype was characterized by histological assessment of aortic sinus atheroma, soluble markers, blood pressure and echocardiography. To place the cardiovascular assessments in the context of intervention effects on bone, structural changes at the tibia were assessed by microtomography.

**Results:**

Vitamin D deficient diet induced significant reductions in plasma vitamin D (p<0.001), trabecular bone volume (p<0.01) and bone mineral density (p<0.005). These changes were accompanied by an increase in calcification density (number of calcifications per mm^2^) of von Kossa-stained aortic sinus atheroma (461 versus 200, p<0.01). Paricalcitol administration suppressed parathyroid hormone (p<0.001), elevated plasma calcium phosphate product (p<0.005) and induced an increase in calcification density (472 versus 200, p<0.005) similar to that seen with vitamin D deficiency. Atheroma burden, blood pressure, metabolic profile and measures of left ventricular hypertrophy were unaffected by the interventions.

**Conclusion:**

Vitamin D deficiency, as well as excess, increases atherosclerotic calcification. This phenotype is induced before other measures of cardiovascular pathology associated clinically with vitamin D deficiency. Thus, maintenance of an optimal range of vitamin D signalling may be important for prevention of atherosclerotic calcification.

## Introduction

Lower levels of circulating active vitamin D (calcitriol) or its precursor, 25-hydroxy vitamin D (25(OH)D), predict increased risk of myocardial infarction [1], [2], stroke [3], [4] and cardiovascular death [5], [6]. Measures of cardiovascular disease associated with lower vitamin D levels in cross sectional analyses include endothelial dysfunction [7], coronary calcification [8] and arterial stiffness [9]. Cardiovascular risk factors such as hypertension [10], diabetes [11], [12], obesity [12] and dyslipidemia [13] are also associated with lower vitamin D levels. These observations are reported across strata of vitamin D concentration in community populations, giving rise to debate regarding definitions of vitamin D deficiency or ‘insufficiency’ [14]. However, it remains unclear whether the reported associations reflect residual confounding from nutrition and lifestyle [13], [14]. Interventional data demonstrating benefits of vitamin D supplementation on clinical cardiovascular endpoints are awaited [15], [16].

The conversion of 25(OH)D to calcitriol is performed by the enzyme one-alpha hydroxylase and occurs primarily in the kidney, regulated by parathyroid hormone, phosphate and fibroblast growth factor-23. Calcitriol acts on the nuclear vitamin D receptor (VDR) to stimulate increased intestinal calcium and phosphate uptake and promote bone mineralization. However, the VDR is also expressed in vascular smooth muscle cells [17], endothelial cells [18], macrophages [19] and cardiomyocytes [20]. Concurrent expression of one-alpha hydroxylase in these cell types suggests a possible paracrine role for vitamin D signalling in the cardiovascular system [21].

Vitamin D receptor knockout (VDR^−/−^) mice manifest increased renin secretion, hypertension and left ventricular hypertrophy [22]. Accelerated atherogenesis has also been reported to accompany VDR knockout in atheroma-prone LDL cholesterol receptor deficient mice [23]. However, the VDR^−/−^ phenotype includes growth retardation, marked hyperparathyroidism, alopecia, and severe bone disease [24]. Cardiovascular findings in this genetic model thus may not be relevant to the above clinical observations (described in populations without osteomalacia). We therefore examined the cardiovascular effects of manipulating vitamin D signalling using vitamin D deficient diets and a VDR agonist in atheroma-prone apolipoprotein E knockout (ApoE^−/−^) mice. Specifically, we hypothesized that: 1) dietary vitamin D deficiency increases aortic sinus atheroma burden, atheroma calcification and left ventricular hypertrophy (LVH), and 2) administration of active vitamin D suppresses atheroma formation and LVH.

## Materials and Methods

### Animals and Interventions

ApoE^−/−^ mice on a C57BL/6 background were obtained from a breeding colony maintained in our unit (original source JAX Labs; JAX2052). Eight week-old males were randomly assigned to vitamin D replete (1.5IU/kg) or deficient (no vitamin D) atherogenic diets (20% cocoa butter, 0.2% cholesterol, 0.03% cholate) with standard calcium (1%) and phosphate (0.6%) content (Harlan Teklad, USA). Between weaning and commencement of test diets, a vitamin D replete diet (1.5IU/kg) was used (Special Diet Services, UK). Animals were housed in a controlled 22°C environment with 12h fluorescent light/dark cycle and free access to food and water.

In an initial experiment the effects of 12 weeks of vitamin D deficient versus replete diet on plasma 25(OH)D levels and bone structure were determined (n = 4 animals per group). Following confirmation of meaningful effects of the dietary intervention, a second experiment examined the cardiovascular consequences of dietary vitamin D deficiency induced by a 20 week intervention period.

From each dietary group (vitamin D replete and vitamin D deficient) animals were further randomized to receive the active vitamin D analogue paricalcitol (Abbott) 400 ng/kg or matched vehicle (20% v/v ethanol, 30% v/v propylene glycol, 50% water) by intraperitoneal injection 3× weekly over the same intervention period (giving 4 groups of n = 7–8 per group). This paricalcitol dose has previously been shown to be well tolerated and to correct secondary hyperparathyroidism in partial renal ablation models [25], [26]. After 20 weeks of intervention animals were euthanized under pentobarbitone anaesthesia. All experiments were approved by the University of Sheffield Project Review Committee and conformed to UK Home Office Regulations (Animal Scientific Procedures Act 1986; Project Licence PPL40/3307).

### Blood Pressure Measurements

Tail cuff blood pressure measurements were performed at 2-weekly intervals on 4 animals per intervention group (Visitech 2000, Visitech Systems, USA). Measurements were taken at the same time of day on each occasion at a controlled temperature of 30°C. An initial set of 10 acclimatization readings were performed prior to the collection of measurements for analysis. Results were based on readings from 20 subsequent cuff inflations per animal, with a mean number of 12 successful readings per session. Mean arterial pressure was calculated from each pair of systolic and diastolic readings.

### Plasma Biochemistry

Plasma calcium, phosphate, urea and lipid fractions were measured by automated bioanalyzer (Beckman Coulter DxC). Whole blood fasting glucose concentration was measured using a portable glucometer (Optium Xceed, Medisense, UK) and commercial ELISAs were used to quantify plasma parathyroid hormone (Immutopics, USA), 25(OH)D (Immunodiagnostic Systems, UK), soluble vascular cell adhesion molecule-1 (sVCAM-1) (R and D Systems, USA) and insulin (Crystalchem, USA). Insulin resistance was measured by homeostatic model assessment (HOMA-IR), calculated as fasting plasma insulin (ng/mL)×fasting plasma glucose (mg/dL)/405. The total plasma nitric oxide oxidation product concentration was measured by Sievers analyser (GE Analytical Instruments, UK).

### Bone Microtomography

The effects of dietary manipulation and paricalcitol on bone structure were assessed by high resolution microtomography (Skyscan, Belgium) analysis of the right tibia. Trabecular bone volume and density were determined using image analysis software with images obtained from a 1 mm length of bone extending distally from 0.2 mm beyond the proximal growth plate.

### Tissue Collection and Preparation

Following aspiration of blood by cardiac puncture the vasculature was flushed with phosphate-buffered saline (PBS) and perfusion-fixed by ventricular injection of 10% v/v formalin. Thoracic aortae were dissected free of connecting tissue from the heart to the level of the diaphragm and fixed in 4% w/v paraformaldehyde for 24h, then stored in PBS prior to staining. After fixation in 10% formalin hearts and brachiocephalic arteries were dehydrated and embedded in paraffin wax for section cutting.

### Assessment of Atheroma Burden/Character

Atheroma burden was quantified in 5 evenly spaced 7 µm cross-sections through the aortic sinus at the level of the aortic valves. Sections were stained with alcian blue/Miller’s elastin van Gieson for atheroma quantification, which was expressed as the mean percentage cross sectional area occupied by atheroma; lumen area was calculated from perimeter measurements. Based on our previous results using similar dietary interventions in ApoE^−/−^ mice [27], the group sizes gave 80% power to detect a mean difference of 30% in aortic sinus cross sectional atheroma burden at alpha = 0.05. Additional assessment of atheroma burden was performed in en face preparations of the thoracic aorta stained for lipid with oil red-O. Determination of the percentage acellular atheroma lesion area was performed on sections stained with haematoxylin/eosin. Morphometric analysis software (NIS-elements Br 3.0, Nikon Instruments, USA) was used for all analyses of atheroma burden and character, by a single assessor blinded to the intervention group.

### Assessment of Atheroma Calcification

For quantification of atheroma calcification, aortic sinus sections were stained by von Kossa’s method with 2% w/v silver nitrate and a nuclear fast red counterstain. Number and area of calcifications were measured using automated software (NIS-elements Br 3.0, Nikon Instruments, USA) with a light wavelength threshold set to identify the black positive (silver salt) von Kossa stain. Since a small number of large calcifications dominated the total calcified lesion area measurements, meaningful statistical comparisons of percentage calcified area between groups were not possible. Similarly, comparing the number of plaques classified as globally calcified (such as applied to human post-mortem studies [28]) was not statistically feasible because there were few lesions with large calcifications. Therefore, diffuse atheromatous calcification was examined with the approach adopted by Schmidt et al. of quantifying the number of distinct calcifications (subanalyzed by size ≥/<100 µm^2^) [29], indexed to atherosclerotic lesion area. The total number of calcifications per mm^2^ atheroma area was considered a measure of diffuse calcification density. Percentage calcified area attributable to calcifications <100 µm^2^ was also compared across intervention groups. The large number of calcifications <100 µm^2^, distributed diffusely in all plaques, allowed a more robust statistical comparison of calcification character by these measures.

### Immunohistochemical Analysis of Aortic Roots

For immunohistochemistry of aortic sinus sections endogenous peroxidases were blocked by immersion in 3% v/v hydrogen peroxide in PBS for 10 min. Antigen retrieval was performed with 10% v/v pH6 citrate buffer (Dako, Germany) in water at 95°C for 20 min and sections were then permeabilized with 0.5% v/v triton X-100 (Sigma) for 5 min at room temperature. Incubation in milk buffer for 30 min was used to block nonspecific antibody binding. Following washing in PBS, sections were incubated with primary antibodies to osteopontin (ab8488, Abcam, UK) at 1∶150 dilution overnight at 4°C, then incubated with horseradish peroxidase-conjugated goat anti-rabbit secondary antibody (P0448, Dako, Denmark) at 1∶200 dilution for 30 min. After repeated washing in PBS, complexes were visualized with diaminobenzidine and sections counterstained with Carazzi’s haematoxylin. Staining was quantified by image analysis software (NIS-elements Br 3.0, Nikon Instruments, USA).

### Echocardiography and Left Ventricular Morphology

Transthoracic echocardiography was performed under isofluorane anaesthesia at week 18–19 (n = 5–6 per group) by a single operator blinded to the experimental status of the mice. Short axis views of the left ventricle (LV) were obtained at the mid papillary muscle level (Vevo 770 ultrasound, Visual Sonics, Canada) and fractional area change determined by manual tracing of the LV wall end diastolic and end systolic areas. Ventricular wall and cavity dimensions were assessed with M-mode measurements; ejection fraction was determined from these measurements by automated software (Visual Sonics). Pulse wave doppler at the aortic annulus was used to measure the velocity timed integral of aortic flow, which was multiplied by the LV outflow tract area to calculate stroke volume and cardiac output. Cardiac output was indexed to body weight for each mouse.

Histological analyses of LV morphology and cardiomyocyte size were performed on haematoxylin/eosin-stained 7 µm sections through the left ventricle 500 µm below the inferior edge of the mitral valve. Mean cardiomyocyte area and diameter were determined from measurements on 50 cells in transverse and longitudinal cross section respectively per mouse (image analysis software as above).

### Statistical Analysis

Data are presented as mean ±standard error. Analyses were performed using GraphPad Prism software version 5 (GraphPad, USA). Groups were compared by one-way ANOVA with Bonferroni correction for multiple comparisons.

## Results

### The Effects of Vitamin D Deficient Diet and Paricalcitol on Plasma Biochemistry and Bone Structure

**Figure 1:**
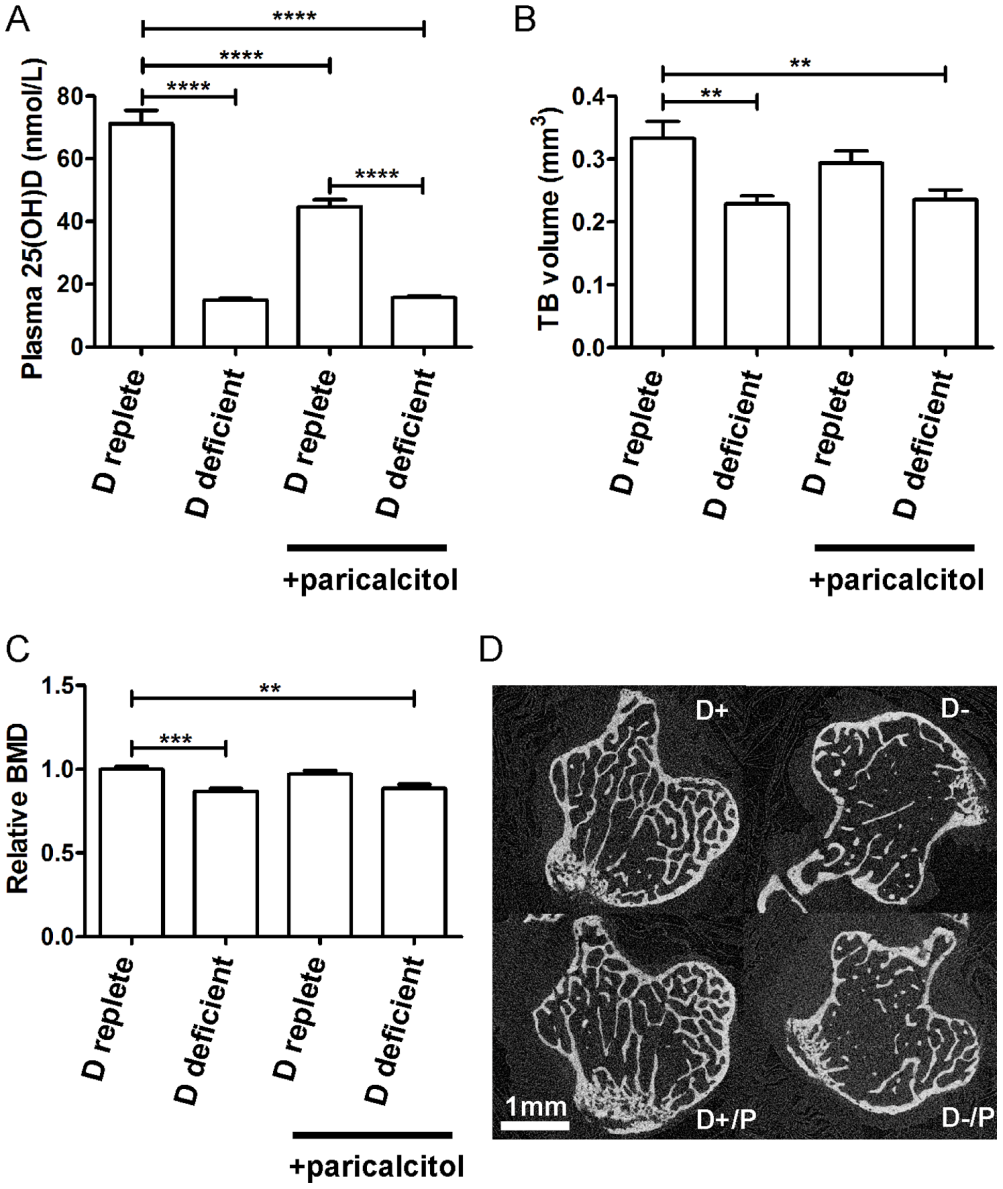
Effects of 20 weeks of vitamin D deficient diet and paricalcitol on 25(OH)D and bone structure. A, plasma 25(OH)D by intervention. B, trabecular bone volume by intervention. C, trabecular bone mineral density relative to that of mice fed a vitamin D replete diet. D, representative microCT images of trabecular bone after 20 weeks of interventions. Groups are labelled according to dietary vitamin D content. Mice that did not receive paricalcitol received matched vehicle. D+, vitamin D replete diet; D-, vitamin D deficient diet; D+/P, vitamin D replete diet with paricalcitol, D−/P, vitamin D deficient diet with paricalcitol. n = 7–8 per group, data presented as mean (SEM). *p<0.05, **p<0.01, ***p<0.005, ****p<0.001. BMD, bone mineral density; TB, trabecular bone.

Feeding a vitamin D deficient diet induced significant reductions in plasma 25(OH)D levels, trabecular bone volume and bone mineral density (Figure 1). These changes were apparent by 12 weeks of dietary intervention (Figure S1), confirming that the 20 week intervention gave a meaningful period of exposure to vitamin D deficiency. Vitamin D deficient diet did not change plasma calcium or phosphate levels (Table 1). However, administration of paricalcitol caused a significant increase in plasma calcium concentration and calcium x phosphate product, accompanied by suppression of parathyroid hormone. When given to mice on a vitamin D replete diet paricalcitol also reduced plasma 25(OH)D levels, consistent with negative feedback induction of 25(OH)D catabolism [30]. Despite the increase in plasma calcium induced by administration of paricalcitol to animals with dietary vitamin D deficiency, trabecular bone changes were not reversed.

**Table 1:** Effects of vitamin D deficient diet and paricalcitol on calcium, phosphate and parathyroid hormone.

	Vit D replete plusvehicle	Vit D deficient plus vehicle	Vit D replete plus paricalcitol	Vit D deficient plus paricalcitol
Calcium (Ca), mmol/L	2.33 (0.02)	2.31 (0.03)	2.72 (0.10)*	2.53 (0.05)*
Phosphate (Pi), mmol/L	2.37 (0.16)	2.32 (0.12)	2.67 (0.13)	2.86 (0.15)
Ca x Pi product, mmol^2^/L^2^	4.91 (0.70)	5.36 (0.31)	7.30 (0.57)*	7.25 (0.45)*
PTH, ng/L	165 (17)	194 (16)	77 (7)†	68 (3)†

n = 7–8 per group, data are given as mean (SEM).

*p<0.005 vs. D replete vehicle, †p<0.001 vs. D replete vehicle. PTH, parathyroid hormone.

### Vitamin D Manipulation does not Affect Blood Pressure, Nitric Oxide Metabolites or Metabolic Profile

**Figure 2:**
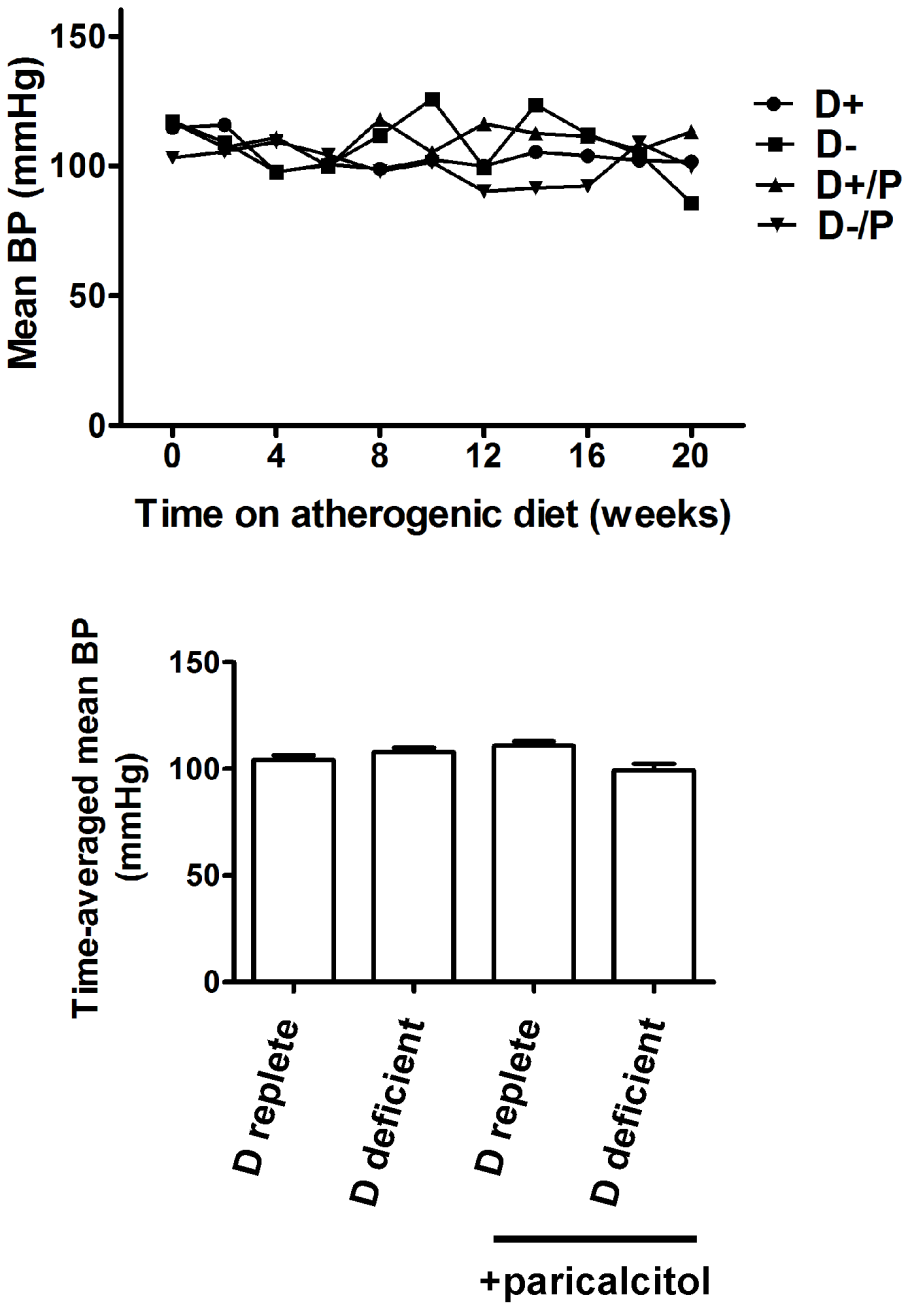
Vitamin D manipulation does not affect blood pressure in ApoE^−/−^ mice. A, mean tail cuff blood pressure by week. B, time-averaged mean blood pressures. n = 4 per group, data presented as mean (SEM). D+, vitamin D replete diet; D-, vitamin D deficient diet; D+/P, vitamin D replete diet with paricalcitol, D−/P, vitamin D deficient diet with paricalcitol.

Manipulation of vitamin D status by feeding a vitamin D deficient diet or the administration of paricalcitol resulted in significantly lower average chow consumption, but did not significantly change the lipid profile, fasting glucose, insulin resistance or body mass index (Table 2). Total plasma nitric oxide metabolites were not suppressed by dietary vitamin D deficiency nor significantly increased by paricalcitol administration. Soluble VCAM-1 levels were also not significantly different between groups. Tail cuff systolic, diastolic and mean blood pressure did not differ significantly by intervention at any stage (Figure 2).

**Table 2:** Effects of vitamin D deficient diet and paricalcitol on metabolic profile.

	Vit D replete plusvehicle	Vit D deficient plus vehicle	Vit D replete plus paricalcitol	Vit D deficient plus paricalcitol
Cholesterol, mmol/L	23.5 (1.8)	20.7 (0.9)	27.2 (1.6)	24.9 (3.0)
HDL cholesterol, mmol/L	7.0 (0.4)	6. 9 (0.4)	7.9 (0.8)	7.5 (0.9)
LDL cholesterol, mmol/L	15.5 (1.5)	13.2 (0.5)	18.9 (1.3)	16.8 (2.6)
Triglyceride, mmol/L	1.0 (0.2)	1.2 (0.2)	1.1 (0.1)	1.0 (0.1)
Urea, mmol/L	8.8 (0.5)	9.9 (0.4)	9.4 (0.8)	8.6 (0.4)
Chow consumption, g/day	3.0 (0.0)	2.6 (0.0)†	2.6 (0.0)†	2.7 (0.0)#
Fasting glucose, mmol/L	11.9 (0.5)	12.7 (0.8)	11.8 (0.8)	11.6 (0.8)
HOMA-IR	0.18 (0.03)	0.24 (0.03)	0.25 (0.06)	0.24 (0.05)
NOx metabolites, nmol/ml	6.5 (1.7)	6.6 (1.4)	6.3 (0.9)	10.8 (3.3)
sVCAM, ng/ml	17.9 (0.6)	19.3 (0.9)	20.3 (0.6)	19.4 (0.5)
Final weight, g	31.8 (1.3)	30.0 (1.0)	32.3 (0.7)	29.5 (0.8)
Body length, mm	177.8 (1.4)	178.3 (2.2)	182.0 (0.8)	176.8 (1.2)
Final BMI, kg/m^2^	0.94 (0.04)	0.90 (0.03)	0.90 (0.02)	0.90 (0.01)

n = 7–8 per group, data are given as mean (SEM).

# p<0.01 vs. D replete vehicle, †p<0.001 vs. D replete vehicle. NOx, nitric oxide; sVCAM, soluble vascular cell adhesion molecule.

### Atheroma Calcification is Increased by Dietary Vitamin D Deficiency but Atheroma Burden is Not

**Figure 3:**
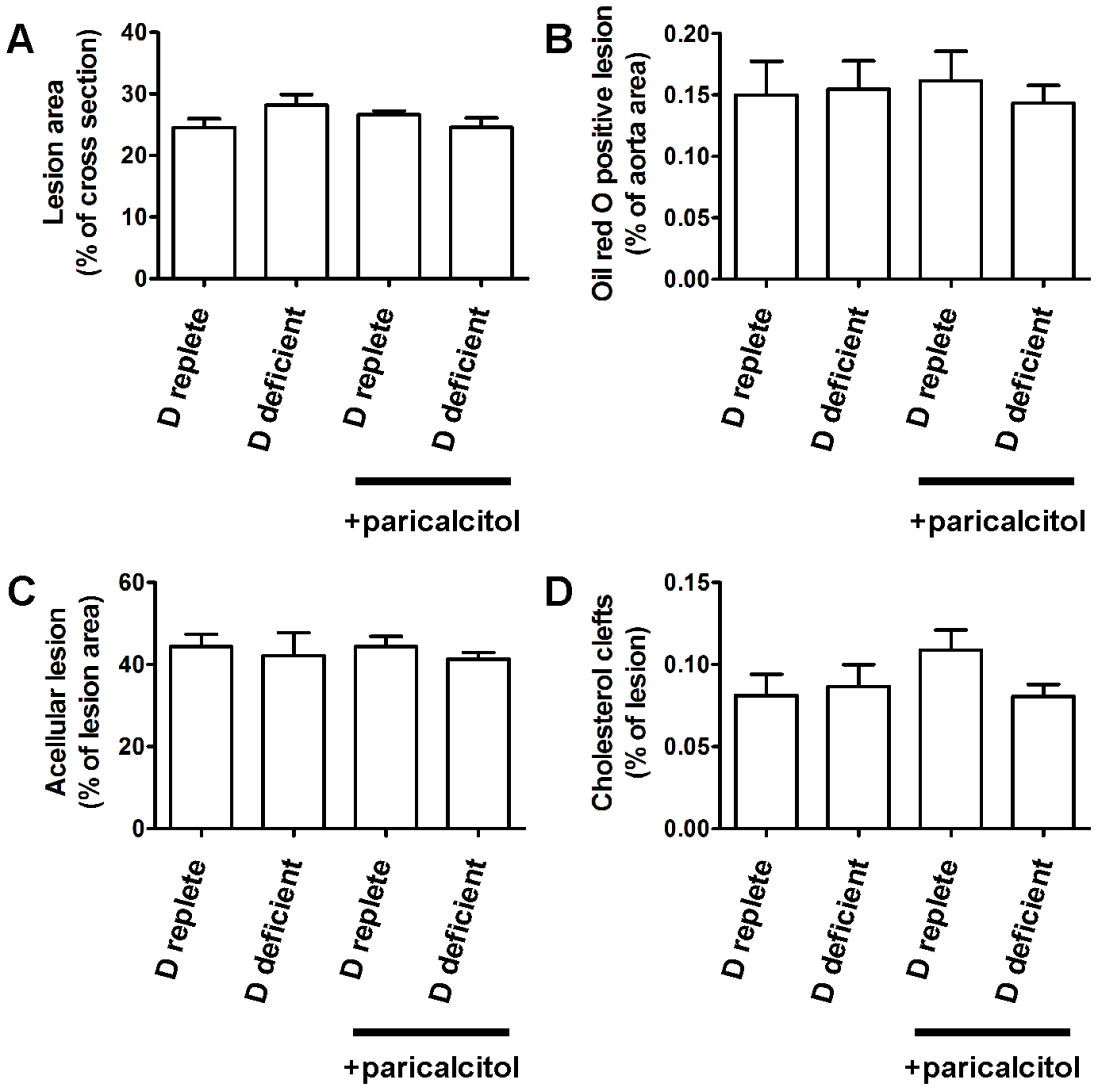
Vitamin D manipulation does not affect atheroma burden, cellularity or lipid cleft area. A, aortic sinus cross sectional atheroma burden. B, thoracic aorta en face atheroma burden. C, percentage of acellular atherosclerosis. D, percentage atheroma cross sectional area occupied by lipid clefts. n = 7–8 per group, data presented as mean (SEM).

Atheroma burden measured in cross sections at the aortic sinus or in en face preparations of the thoracic aorta was not significantly different between groups (Figure 3A,B and Figure S2). Atheroma cellularity and the percentage area occupied by lipid clefts were also unaffected by vitamin D manipulation (Figure 3C,D).

**Figure 4:**
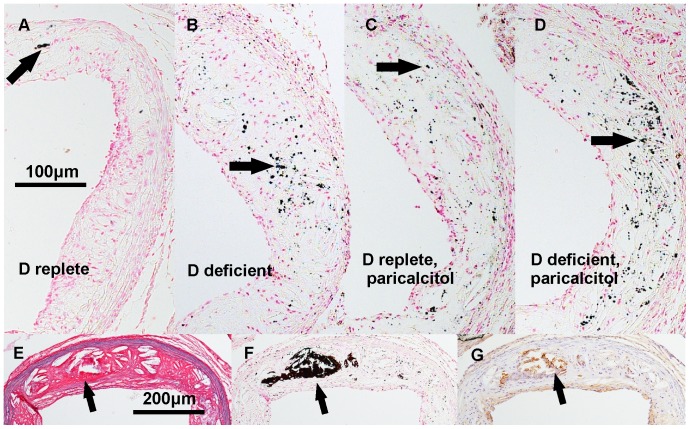
Patterns of atheromatous calcification in ApoE^−/−^ mice. A–D, representative atherosclerotic lesions demonstrating diffuse calcification (stained black by von Kossa method, arrows) from mice fed vitamin D replete and vitamin D deficient diets and coadministered vehicle (A and B respectively) or paricalcitol (C and D respectively). E–G, sequential sections stained with Miller’s elastin Van Gieson, von Kossa method and osteopontin-specific antibody respectively, demonstrating a necrotic area of lesion (E) containing a large calcification (F) with associated osteopontin staining (G) (arrows), taken from a vitamin D replete mouse administered paricalcitol.

**Figure 5:**
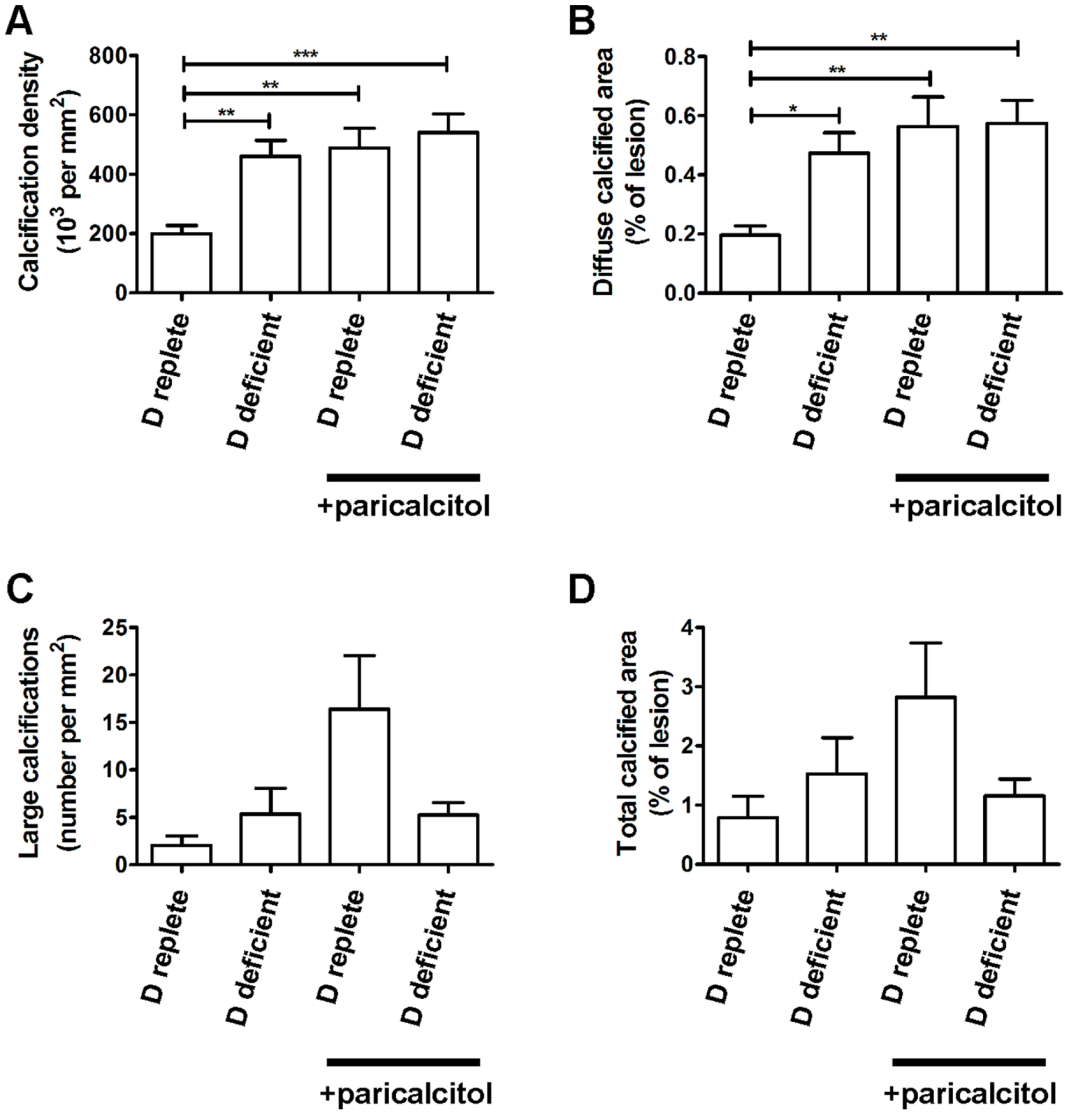
Vitamin D deficiency and excess similarly increase atherosclerotic calcification. A, number of calcifications normalized to lesion area. B, percentage calcified lesion area due to calcifications <100 µm^2^. C, number of very large calcifications (≥100 µm^2^). D, total calcified area due to diffuse and large calcifications. n = 7–8 per group, data presented as mean (SEM). *p<0.05, **p<0.01, ***p<0.005.

There was a significant increase in the diffuse calcification of aortic sinus atherosclerosis assessed by von Kossa staining in mice fed a vitamin D deficient diet or administered paricalcitol (Figures 4 and 5). Small calcifications (<100 µm^2^) were present diffusely throughout the atherosclerotic lesions (Figure 4A–D); a small number of much larger calcifications were also present in association with necrotic regions in all groups (Figure 4E–G). The total number of calcifications per mm^2^ lesion area was more than doubled in mice fed a vitamin D deficient diet or administered paricalcitol compared to mice fed a vitamin D replete diet. Total percentage calcified lesion area was also greater in vitamin D-deficient mice and mice administered paricalcitol, but this was not statistically significant, reflecting the small number of very large calcifications dominating the total calcified area measurement. The number of large calcifications (≥100 µm^2^) was also nonsignificantly greater in atheroma from vitamin D deficient and paricalcitol-treated mice versus D-replete diet vehicle-treated mice. When the percentage calcified lesion area attributable to the diffuse small lesions (<100 µm^2^) was considered, this was significantly greater for vitamin D deficient mice (p<0.05 vs. mice on vitamin D replete diet) and paricalcitol-administered mice (p<0.01 vs. mice on a vitamin D replete diet). The percentage calcified aortic valve area and number of valve calcifications per mm^2^ did not differ significantly between groups (not shown). Immunostaining did not show any significant differences in atheroma or valve osteopontin expression. As previously reported [31], intense staining for osteopontin was evident at sites of dystrophic calcification (Figure 4E–G).

### Left Ventricular Hypertrophy is not Induced by Dietary Vitamin D Deficiency in ApoE^−/−^ Mice

**Figure 6:**
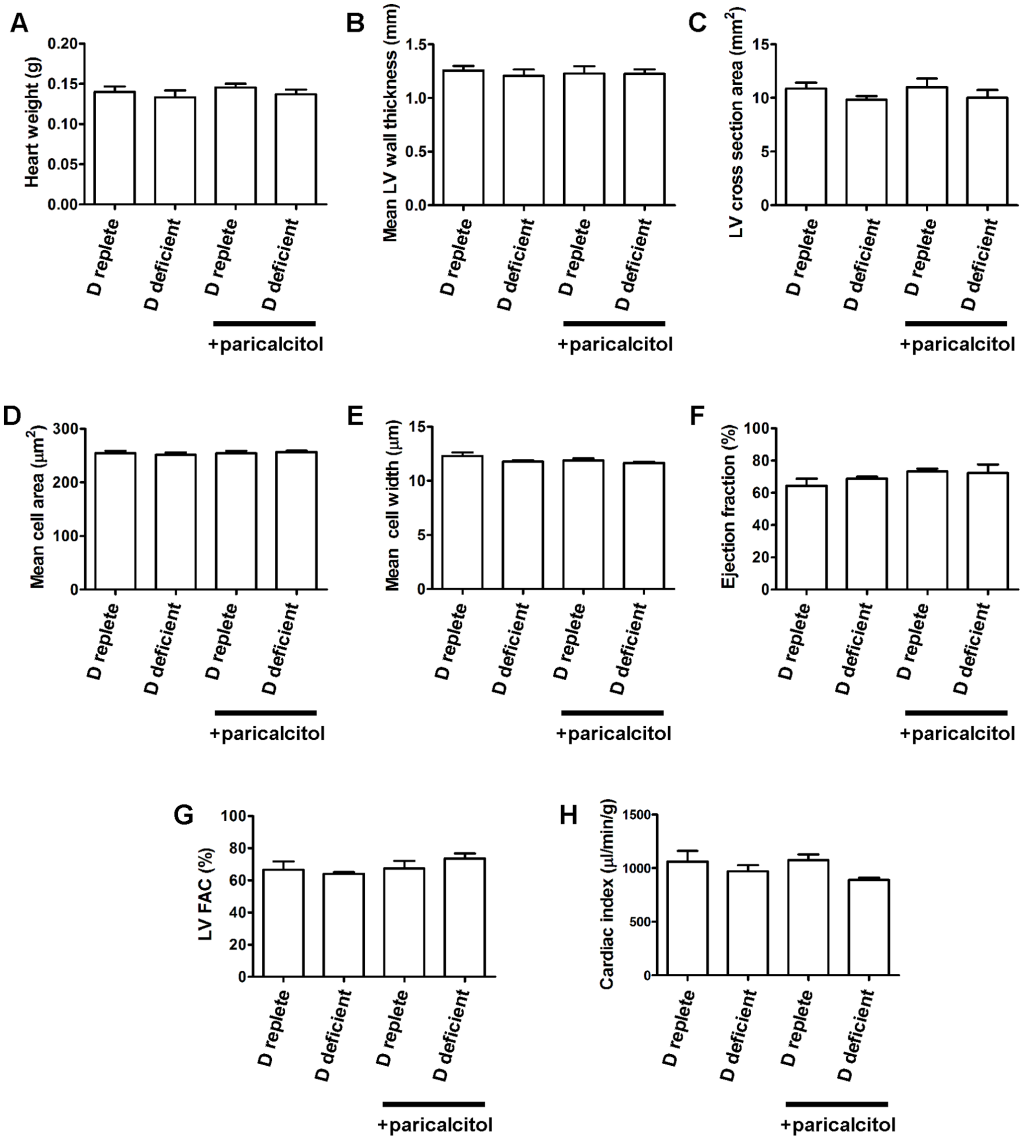
Vitamin D deficiency does not induce left ventricular hypertrophy in ApoE^−/−^ mice. A, heart weights by intervention. B and C, mean LV wall thickness and LV cross sectional area measured histologically. D and E, cardiomyocyte cross sectional area and transverse diameter. F, left ventricular ejection fraction. G, left ventricular fractional area change. H, cardiac index. n = 7–8 per group for histological analyses and weights, n = 5–6 per group for echocardiography. Data presented as mean (SEM).

Cardiac weights did not differ between the intervention groups (Figure 6). Histological assessment of LV morphology including mean LV wall thickness, LV wall cross sectional area, cardiomyocyte transverse area and cardiomyocyte diameter also did not show any differences between groups. Echocardiographic functional parameters of ejection fraction, fractional area change and cardiac index were similarly unchanged.

## Discussion

Despite the large body of observational data linking lower vitamin D levels to cardiovascular disease, interventional evidence for a causative role of lower vitamin D levels in cardiovascular pathology is relatively scarce. We report the novel finding that dietary vitamin D deficiency induces a substantial increase in diffuse atherosclerotic calcification. The extent of this increase is similar to that induced by the administration of a VDR agonist dose sufficient to raise the plasma calcium phosphate product, a recognised stimulus to arterial calcification in nonatheromatous animal models [32]. The increase in diffuse atherosclerotic calcification induced by vitamin D deficiency occurred at a degree of deficiency where no increases in atheroma burden, metabolic derangement or left ventricular hypertrophy were evident.

These results with regard to calcification are consistent with clinical observational data. Lower 25(OH) vitamin D levels were an independent predictor of coronary artery calcification in an asymptomatic population [33] and polymorphisms in the vitamin D regulatory gene CYP24A1 have been associated with coronary calcification in a cross-sectional analysis [34]. However, standard clinical assessments do not distinguish between calcification differences due to changes in atheroma character, atheroma burden and nonatherosclerotic medial calcification. To our knowledge, whether vitamin D status predicts atheroma calcification on intravascular ultrasound has not been reported.

Our findings support and extend those of a recent study reporting that a low vitamin D diet increased the calcified area of aortic sinus sections in LDL receptor knockout (LDLR^−/−^) mice [29]. That report did not, however, determine whether the location of the increased calcification was in atheroma or the aortic valves. Also consistent with our findings, Mathew et al. reported suppression of aortic atherosclerotic calcification by low doses of active vitamin D in partially nephrectomised LDLR^−/−^ mice, suggesting restoration of a calcification-inhibitory effect of VDR signalling [26]. High doses of paricalcitol increased aortic calcium content in their model, as in our study, consistent with there being an optimum range of VDR signalling for calcification prevention.

Our findings are also consistent with some evidence for a beneficial effect of VDR signalling in the prevention of arterial *medial* calcification (as occurs in kidney disease). Vitamin D receptor agonists (at non-hypercalcemic/hyperphosphatemic doses) have been shown to suppress medial calcification in a high-phosphate diet partial renal ablation mouse model [35]. Whether dietary vitamin D deficiency accelerates arterial medial calcification is unknown; nonatherosclerotic medial calcification was not prominent in our model and requires induction by precipitating factors in animal models (such as renal ablation combined with high phosphate intake [35]). However, some evidence of a bi-modal relationship between vitamin D status and vascular calcification score has been reported in pediatric dialysis patients [36].

Addition of paricalcitol or calcitriol to vascular smooth muscle cell-macrophage cocultures has previously been demonstrated to inhibit phosphate-induced smooth muscle cell calcification via a mechanism involving stimulation of macrophage osteopontin expression [37]. We did not find any difference in atherosclerotic lesion osteopontin expression accompanying vitamin D manipulation in our model. However this does not mean that osteopontin is not responsible for mediating anticalcific effects of vitamin D; osteopontin is expressed at sites of vascular calcification so may be both a marker and inhibitor of calcification processes [31]. Schmidt et al. reported increased osteopontin expression accompanying the increased calcification induced by vitamin D deficiency [29].

The type of vitamin D therapy as well as the dose could be clinically important for calcification prevention. Activated vitamin D (calcitriol) or analogues (such as paricalcitol) act systemically to increase intestinal calcium and phosphate uptake, bypassing the regulatory control of renal vitamin D activation. As seen in our model and others [26], [32], the resulting increase in plasma calcium and phosphate levels may be accompanied by an increase in vascular calcification. Replenishing instead the precursor, 25(OH)D, could restore paracrine vitamin D signalling in cardiovascular tissue without necessarily raising plasma calcium phosphate product. This is of particular clinical relevance in the setting of chronic kidney disease, where a deficiency of renal vitamin D activation is commonly accompanied by nutritional vitamin D deficiency [38]. Our findings suggest that correcting 25(OH) vitamin D deficiency might be beneficial for the prevention of vascular calcification in these patients. Treating with an active vitamin D analogue *without* replenishing 25(OH)D theoretically risks combining the adverse consequences of increased calcium phosphate product with persisting deficiency of paracrine vitamin D signalling. In our model, combining paricalcitol administration with 25(OH)D deficiency did not result in a greater degree of atherosclerotic calcification than either intervention alone. However, although the dose of paricalcitol we employed was sufficient to raise calcium phosphate product, it did not restore structural bone changes resulting from 25(OH)D deficiency. Bone marrow stromal cells express 1-alpha hydroxylase [39] so our findings may reflect an important role for local 25(OH)D activation in maintaining bone structure. To our knowledge there are no clinical studies examining differential effects on bone structure of 25(OH)D replacement versus active vitamin D administration in the setting of 25(OH)D deficiency.

As in the LDLR^−/−^ model of Schmidt et al. [29], we found no significant increase in aortic atherosclerosis burden in ApoE^−/−^ mice fed a vitamin D-deficient diet. This is in contrast to the previously reported acceleration of atherogenesis in LDLR^−/−^ mice crossed with VDR^−/−^ mice [23], perhaps reflecting a lesser degree of attenuation of vitamin D signalling by our dietary manipulation. The severe phenotype of VDR^−/−^ mice makes it difficult to translate accompanying cardiovascular findings to clinical associations of mild vitamin D deficiency/insufficiency. However, Weng et al. recently reported an increase in atheroma burden induced by dietary vitamin D deficiency in LDLR^−/−^ and ApoE^−/−^ models [40]. Again, the contrast with our findings may be a consequence of the degree of vitamin D manipulation; Weng et al. commenced vitamin D deficient diets at weaning whereas we commenced the D-deficient diet at 8 weeks and Schmidt et al. used a diet that was not completely D-deficient. Nevertheless, both our intervention and that of Schmidt et al. achieved relative reductions in 25(OH)D greater than those associated with adverse cardiovascular outcomes clinically.

Conflicting results have also been reported regarding the effects of VDR agonists on atherosclerosis burden. Takeda et al. found a significant reduction in aortic sinus atheroma with the administration of oral calcitriol to ApoE^−/−^ mice [41]. In contrast, Becker et al. found no benefit of intraperitoneal calcitriol or paricalcitol administration in ApoE^−/−^ mice, but an attenuation of uninephrectomy-accelerated atherogenesis with paricalcitol [42]. We used a greater paricalcitol dose than Becker et al., but also found no suppression of atherogenesis in a non-nephrectomised model. It is possible that too high a dose of VDR agonist nullifies potential atherosuppressive benefits of increased VDR signalling. Unlike our regime, the calcitriol dose administered by Takeda et al. had no effect on plasma phosphorus and calcium concentrations. We and others have previously demonstrated that greater dietary phosphorus exposure accelerates atherogenesis in ApoE^−/−^ mice [27], [43]. Increased intestinal phosphorus uptake accompanying excessive VDR agonist use may thus counteract atheroprotective benefits.

The absence of left ventricular histological or echocardiographic changes induced by vitamin D deficiency in this study contrasts with findings from global and cardiomyocyte-specific VDR^−/−^ mice [20], [24]. As with the conflicting atherosclerosis data, this may reflect differences in the degree of attenuation of VDR signalling.

A strength of our study is the simultaneous characterisation of the effects of dietary vitamin D deficiency on bone and the cardiovascular system. Observational clinical data associate cardiovascular outcomes with lower 25(OH)D levels across a range that is also associated with significant but small reductions in bone mineral density [1], [5], [44]. In our model dietary vitamin D deficiency induced relative changes in bone mineral density by 12 weeks (i.e. 8 weeks before the cardiovascular assessments) greater than those associated with variation in vitamin D levels in community populations [44]. This suggests that the degree of vitamin D deficiency attained by our intervention approach was sufficiently severe to be physiologically relevant. Consequently, cardiovascular pathology induced in more severe models of vitamin D deficiency may not relate to clinical observations, though there may of course be species differences in tissue-specific susceptibility to vitamin D deficiency.

Our model suggests that increased diffuse atherosclerotic calcification is an earlier sequel of vitamin D-deficiency than adverse metabolic profile, hypertension and lower nitric oxide levels. The relevance of this increase to the association of lower vitamin D levels with cardiovascular outcomes is unclear. Further work is needed to determine the underlying mechanism(s) and consequences of this phenomenon. Importantly, cardiovascular benefits of vitamin D supplementation are currently being investigated in a large clinical trial [45].

## Supporting Information

Figure S1 **Effects of 12 weeks vitamin D deficient diet on plasma 25(OH)D and bone structure.** A, plasma 25(OH)D by intervention. B, trabecular bone volume by intervention. C, trabecular bone mineral density relative to that of mice fed a vitamin D replete diet. D, representative microCT images of trabecular bone after 12 weeks of dietary intervention. n = 4 per group for, data presented as mean (SEM). *p<0.05, **p<0.01, ****p<0.001. BMD, bone mineral density; TB, trabecular bone.
(TIF)

Figure S2 **Sample images of atheroma characterisation.** A, aortic sinus atheroma stained with Miller’s elastin-van Gieson. B, thoracic aorta stained for lipid with oil red O. C, Millers’ elastin van Gieson-stained section with example lipid clefts indicated by arrows. D, haematoxylin and eosin-stained lesion with some areas of acellularity marked.
(TIF)
