# Supplementary figures and images for: Vitamin D Deficiency and Exogenous Vitamin D Excess Similarly Increase Diffuse Atherosclerotic Calcification in Apolipoprotein E Knockout Mice
Source: PLoS One. 2014 Feb 19;9(2):e88767. doi: 10.1371/journal.pone.0088767 (PMC3929524; doi:10.1371/journal.pone.0088767)

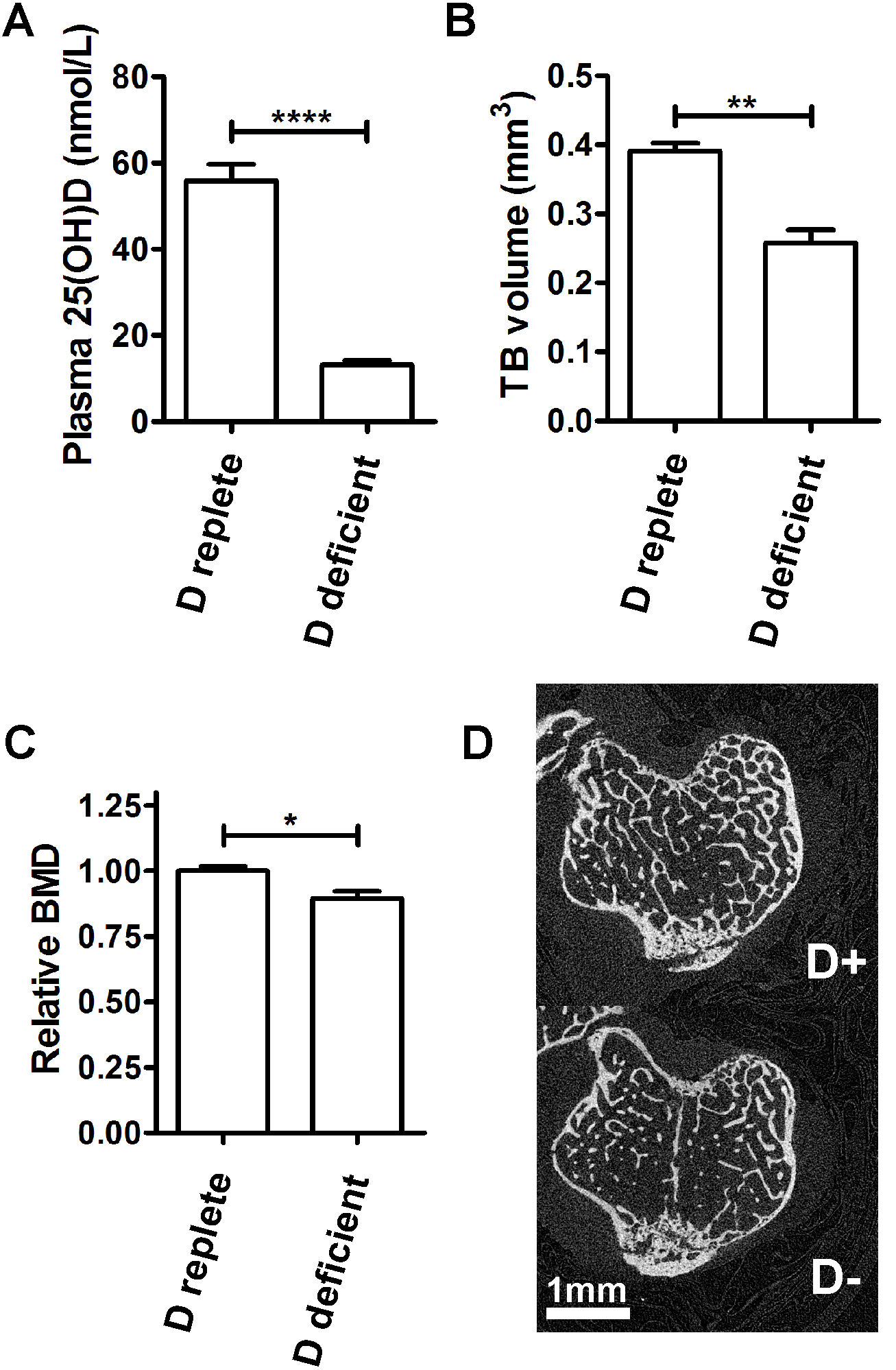

Supplement: Figure S1 — Effects of 12 weeks vitamin D deficient diet on plasma 25(OH)D and bone structure. A, plasma 25(OH)D by intervention. B, trabecular bone volume by intervention. C, trabecular bone mineral density relative to that of mice fed a vitamin D replete diet. D, representative microCT images of trabecular bone after 12 weeks of dietary intervention. n = 4 per group for, data presented as mean (SEM). *p<0.05, **p<0.01, ****p<0.001. BMD, bone mineral density; TB, trabecular bone. (TIF) [file pone.0088767.s001.tif]

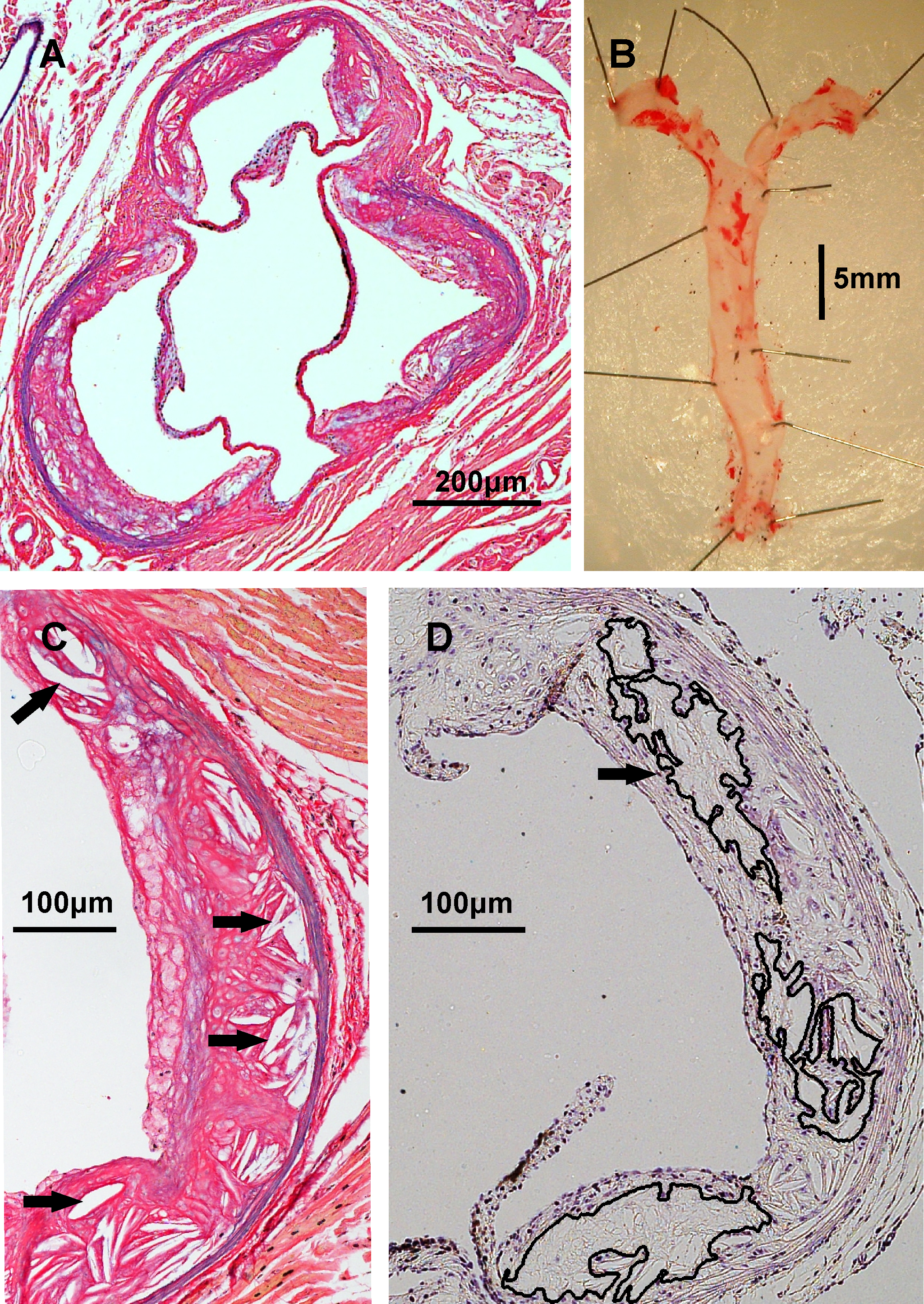

Supplement: Figure S2 — Sample images of atheroma characterisation. A, aortic sinus atheroma stained with Miller’s elastin-van Gieson. B, thoracic aorta stained for lipid with oil red O. C, Millers’ elastin van Gieson-stained section with example lipid clefts indicated by arrows. D, haematoxylin and eosin-stained lesion with some areas of acellularity marked. (TIF) [file pone.0088767.s002.tif]
